# Referring Service Effect on Muscle Biopsy Diagnosis and Management in Myopathy

**DOI:** 10.7759/cureus.2800

**Published:** 2018-06-13

**Authors:** Steven O Tenny, Kyle P Schmidt, Kenneth A Follett

**Affiliations:** 1 Neurosurgery, University of Nebraska Medical Center, Omaha, USA

**Keywords:** muscle biopsy, referring service, diagnostic yield, therapeutically useful, therapeutic yield

## Abstract

Introduction

Requests for muscle biopsy for evaluation of myopathy originate from a variety of different specialties. It is unknown whether the specialty of the referring service affects the likelihood of diagnostic biopsy or the therapeutic usefulness of a biopsy.

Methods

We reviewed 106 consecutive muscle biopsies requested by healthcare providers in neurology, rheumatology, family medicine, oncology, and gastroenterology. We tested for an association between referring service and whether the biopsy yielded a definitive pathology result or provided therapeutically useful results.

Results

Half of the biopsies (49%) returned definitive pathology and 88% of the biopsies provided information that contributed to therapeutic decisions. The diagnostic yield for muscle biopsies referred by different services was not statistically significant (p-value 0.1344) nor was the therapeutic yield statistically significant for muscle biopsies referred by different services (p-value 0.5525).

Discussion

The specialty of the service that requests a muscle biopsy does not influence the likelihood of obtaining a definitive pathological diagnosis or therapeutically useful information. Other factors may be more important in determining the likelihood of obtaining a clinically useful biopsy result.

## Introduction

Myopathies often present in a gradual manner with symptoms including weakness, myalgia, and lethargy [1]. Primary causes of myopathies include muscular dystrophy, inflammatory conditions, metabolic disease or congenital myopathy. Secondary causes of myopathies occur due to neurogenic disease [2]. Work up for myopathy includes history, physical examination, imaging, laboratory, and electromyographic testing [3-5].

If a diagnosis cannot be established with other clinical means then the gold standard is a muscle biopsy [6-7]. A muscle biopsy has an estimated diagnostic yield of 30%–70% [3, 6, 8-11]. Muscle biopsy is a relatively minor procedure, but it has inherent risks including hematoma, muscle herniation, infection, wound dehiscence, pain, and weakness, in addition to the risk of being non-diagnostic [4, 8]. Therefore, care should be taken to identify and refer those patients who are likely to derive therapeutic benefit from the procedure while refraining from referring those patients who are unlikely to benefit. A variety of factors may play a role in determining the predictive value of a diagnostic muscle biopsy [1, 3, 5-8, 12] or therapeutic impact of the biopsy affecting treatment [6, 8, 10]. To date, the literature has not examined the effect of referring service on the usefulness of a muscle biopsy. Our goal is to review consecutive referrals for muscle biopsy and determine if there is an association between the referring service requesting the muscle biopsy and the likelihood of definitive pathologic diagnosis or therapeutic usefulness of the muscle biopsy.

## Materials and methods

We performed a retrospective chart review at a single institution. Inclusion criteria were patients age 19 and older with a muscle biopsy between January 1, 2012, and July 1, 2016. Muscle biopsies were identified as encounters coded with Common Procedural Terminology (CPT, AMA® 2016) code 20200 (superficial muscle biopsy) or 20205 (deep muscle biopsy). All biopsies were performed by one of five experienced neurosurgeons. For each biopsy, two segments of muscle tissue were obtained from the same muscle using muscle biopsy clamps. The specimen was sent directly to the pathology lab for immediate processing. The biopsies were examined by a trained neuropathologist.

Charts were abstracted to identify patient gender, age, referring service, and pathologic results and therapeutic implications of the biopsy. Prior to project initiation, Institutional Review Board (IRB) approval was obtained (IRB 502-16-EX).

The referring service was the documented service who consulted neurosurgery for the muscle biopsy. The results of the muscle biopsies were dichotomized as providing a definite pathologic result or not providing a definite pathologic result. Definite pathologic results were those that named a single, specific disease process identified in the biopsied muscle tissue. All biopsies which did not name a single, specific disease process were categorized as non-definitive pathologic results.

Therapeutic usefulness of the biopsies was assessed by reviewing chart documentation up to one year after the pathology report was available. If chart documentation indicated that the biopsy result was considered as part of the reasoning for treatment decisions, then the muscle biopsy was considered to have therapeutic usefulness. If the above criterion was not met, then the muscle biopsy was considered to not have therapeutic usefulness. Muscle biopsies which did not report definitive pathology could still have therapeutic usefulness if the pathology report was used to guide subsequent therapy, e.g., a non-diagnostic biopsy may provide therapeutically useful information by ruling out some disorders.

The categorical variables of referring service, definite pathology result (pathologic yield) and therapeutic usefulness (therapeutic yield) were analyzed in a n x c table. Two sets of comparisons were made. First, the referring services were compared to each other based on the pathologic yield of the muscle biopsies they referred to determine whether there was a statistically significant difference in the pathologic yield of muscle biopsies between referring service. Secondly, we compared referring services based on the therapeutic yield of the muscle biopsies they requested to determine whether there was a statistically significant difference in the therapeutic yield of the muscle biopsies between referring services. All analyses for the two n x c tables were done using the Fisher-Freeman-Halton exact test. A level of significance was pre-specified as p < 0.05. Analysis was carried out using SAS versions 9.4 (SAS Institute Inc., Cary, NC).

## Results

A total of 106 consecutive muscle biopsies were included in the study. Muscles biopsied were vastus lateralis (32%), quadriceps not otherwise specified (25%), deltoid (17%), biceps (8%), rectus femoris (4%), paraspinal (3%), triceps (3%), vastus medialis (3%), gluteus (2%), gastrocnemius (1%), hamstring (1%), and trapezius (1%).

Patient ages ranged from 21 to 86 years old with a mean of 57 years and standard deviation 17 years. Fifty-five percent (58/106) of patients were males. Of the 106 biopsies, 72 were requested by neurology, 31 by rheumatology, and one each by family medicine, oncology, and gastroenterology.

Of the 106 muscle biopsies, 52 had a definite pathologic abnormality reported (49%) and 54 had non-definitive pathology reported by the pathologist (51%). Biopsies had therapeutic usefulness in 93 cases (88%) and no therapeutic implications in 13 biopsies (12%).

Presence or absence of definitive pathology was not related significantly to referring service, i.e., no specific referring service was any more or less likely to refer patients who had definitive pathology (p-value 0.1344) (Table 1). Similarly, presence or absence of therapeutically useful information was not related significantly to the specialty of the referring service, i.e., no specific referring service was more or less likely than any other service to refer patients who ultimately had therapeutically useful biopsies (p-value 0.5525) (Table 2).

**Table 1:** Presence or absence of definitive pathology on biopsy versus referring service

Referring Service	Definitive Pathology Present (n)	Definitive Pathology Not Present (n)	Total (n)
Neurology	32	40	72
Rheumatology	19	12	31
Family Medicine	0	1	1
Oncology	1	0	1
Gastroenterology	0	1	1
Total	52	54	106

**Table 2:** Therapeutic usefulness of biopsy versus referring service

Referring Service	Therapeutically Useful (n)	Not Therapeutically Useful (n)	Total (n)
Neurology	61	11	72
Rheumatology	29	2	31
Family Medicine	1	0	1
Oncology	1	0	1
Gastroenterology	1	0	1
Total	93	13	106

The majority of referrals were from neurology (68%) and rheumatology (29%) with few coming from the other specialties. To determine whether the small number of referrals from non-neurology specialties limited the power of our study to detect a difference between specialties, we performed a post-hoc analysis comparing neurology to all other referring services combined. Fisher exact test analysis did not demonstrate a statistically significant difference for definitive pathology (p-value 0.2402) or therapeutic usefulness (p-value 0.2877) between neurology and all other referring specialties. Similarly, a post-hoc Fisher exact test analysis comparing rheumatology to all other referring services combined did not yield a statistically significant difference for definitive pathology (p-value 0.1593) or therapeutic usefulness (p-value 0.4040).

## Discussion

The diagnostic yield of muscle biopsies, as reported in the literature, ranges from 30-70% [3, 6, 8-11]. Careful preoperative consideration of factors that might improve diagnostic yield would be in the best interests of patients by facilitating identification of patients who are likely to benefit from biopsy and excluding from biopsy procedures those patients who are unlikely to have a diagnostic result. One such factor might be the specialty of the referring service that requests biopsy. To our knowledge, this study is the first to explore the effect of referring service on muscle biopsy diagnostic yield and therapeutic impact.

Several sets of investigators have evaluated preoperative factors that may affect the likelihood of diagnostic pathology result, but only a few provided information about referring service and none evaluated the impact of referring service. For instance, Lai et al. [6] explored preoperative factors which may affect obtaining diagnostic muscle biopsy results or clinically relevant pathology but did not identify the referring service other than stating all biopsies were referred by general physicians or specialists. Reynolds et al. [8] evaluated only referrals from neurology services and Claussen et al. [13] reported that 12% of their referrals were from neurology services and the remainder were from primary care physicians. Other authors who studied factors that influence diagnostic yield did not provide information about specialty of referring services [5, 7, 9].

We were unable to find any study looking specifically at whether the specialty of a referring service affects the diagnostic yield or therapeutic usefulness of a muscle biopsy. We felt this question warranted scrutiny in order to maximize the likelihood that a patient would have a diagnostically or therapeutically valuable biopsy. Although some may argue all patients with myopathy should be seen by a specialist prior to referral for biopsy (e.g., a neuromuscular specialist), published literature reveals widely varying sources of referrals for muscle biopsy [6, 8, 13]. The diversity of sources of referrals for muscle biopsy reflects the heterogeneity of specialties taking care of patients with myopathy and myopathic-like processes. Published reports describe mainly what types of pre-biopsy evaluations have been conducted without considering the specialty of the provider who supervised the evaluation [1, 3, 5-8,12, 13]. Different specialties tend to take different approaches to evaluation of myopathies and related processes, so the nature and extent of the preoperative evaluation can vary widely and could be expected to influence the likelihood of obtaining a diagnostically and/or therapeutically useful muscle biopsy result.

We did not observe a statistically significant difference between referring service and likelihood of obtaining definitive diagnostic pathology or therapeutic usefulness, so we conclude that referral to a provider in a specific specialty is not the key driver for muscle biopsy outcome. To the contrary, our findings suggest that healthcare providers in a variety of specialties may be equally proficient in evaluating myopathy and identifying patients who may benefit from muscle biopsy.

Our study has several limitations. As noted in the results, the small number of referrals from specialties other than neurology and rheumatology limits the statistical power of our analysis with respect to those referring sources. All subjects in our study were referred to a tertiary academic center where potentially more complex cases are seen that might be evaluated in the community setting; also, and the tertiary care specialists who evaluate these patients may have approached to preoperative evaluation differently from community colleagues. We limited our analysis to the adult population and do not know whether an evaluation of pediatric patients would have a similar outcome.

## Conclusions

We did not observe an association between the specialty of a service requesting a muscle biopsy and the diagnostic or therapeutic yield of the muscle biopsy. These findings suggest that the referring service for a muscle biopsy may not be the significant factor affecting the diagnostic or therapeutic yield of a muscle biopsy.
